# *N*′-[(1*E*,2*E*)-1-(2-Phenyl­hydrazin-1-yl­idene)-1-(phenyl­sulfon­yl)propan-2-yl­idene]benzohydrazide

**DOI:** 10.1107/S1600536811031953

**Published:** 2011-08-11

**Authors:** Hatem A. Abdel-Aziz, Seik Weng Ng, Edward R. T. Tiekink

**Affiliations:** a Department of Pharmaceutical Chemistry, College of Pharmacy, King Saud University, Riyadh 11451, Saudi Arabia; b Department of Chemistry, University of Malaya, 50603 Kuala Lumpur, Malaysia; c Chemistry Department, Faculty of, Science, King Abdulaziz University, PO Box 80203 Jeddah, Saudi Arabia

## Abstract

The configuration about each C=N bond in the title compound, C_22_H_20_N_4_O_3_S, is *E*. While to a first approximation the phenyl­hydrazin-1-yl­idene and benzohydrazide residues are coplanar, in part due to the presence of an intra­molecular N—H⋯N hydrogen bond, significant twists are evident in the orientations of the hydrazine [N—N—C—C torsion angle = −170.74 (11)°] and benzoyl benzene [N—C—C—C = −21.72 (18)°] rings. The sulfonyl benzene ring occupies a position almost normal to the rest of the mol­ecule [C—S—C—N = −92.28 (10)°]. Centrosymmetric aggregates mediated by pairs of hydrazide–sulfonyl N—H⋯O hydrogen bonds are the predominant packing motif in the crystal. These are connected into linear supra­molecular chains *via* C—H⋯O inter­actions which are, in turn, linked into layers in the *ac* plane *via* C—H⋯π inter­actions. Connections between layers along the *b*-axis direction are of the π–π type and occur between centrosymmetrically related hydrazine-bound benzene rings [centroid–centroid separation = 3.7425 (9) Å].

## Related literature

For background to the biological activity of bis-hydrazones, see: Abdel-Aziz & Mekawey (2009 ▶); Abdel-Aziz *et al.* (2010 ▶).
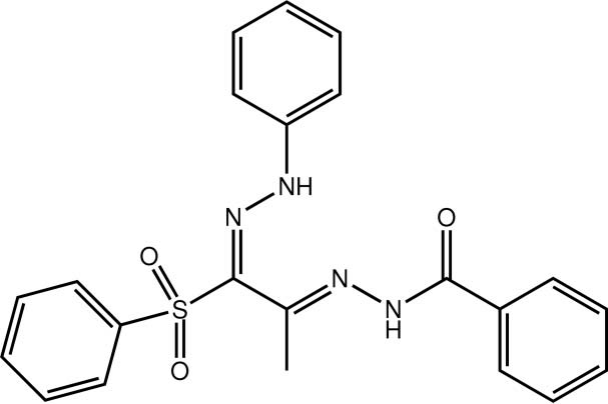


## Experimental

### 

#### Crystal data

C_22_H_20_N_4_O_3_S
*M* *_r_* = 420.48
Triclinic, 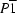

*a* = 8.1609 (3) Å
*b* = 9.6632 (5) Å
*c* = 14.1261 (7) Å
α = 92.027 (4)°
β = 102.822 (4)°
γ = 111.984 (4)°
*V* = 998.55 (8) Å^3^
*Z* = 2
Cu *K*α radiation
μ = 1.72 mm^−1^
*T* = 100 K
0.30 × 0.25 × 0.05 mm

#### Data collection

Agilent SuperNova Dual diffractometer with an Atlas detector
Absorption correction: multi-scan (*CrysAlis PRO*; Agilent, 2010 ▶) *T*_min_ = 0.825, *T*_max_ = 1.000
6674 measured reflections
3942 independent reflections
3658 reflections with *I* > 2σ(*I*)
*R*_int_ = 0.017

#### Refinement

*R*[*F* ^2^ > 2σ(*F* ^2^)] = 0.031
*wR*(*F* ^2^) = 0.085
*S* = 1.04
3942 reflections
280 parameters
H atoms treated by a mixture of independent and constrained refinement
Δρ_max_ = 0.34 e Å^−3^
Δρ_min_ = −0.42 e Å^−3^

### 

Data collection: *CrysAlis PRO* (Agilent, 2010 ▶); cell refinement: *CrysAlis PRO*; data reduction: *CrysAlis PRO*; program(s) used to solve structure: *SHELXS97* (Sheldrick, 2008 ▶); program(s) used to refine structure: *SHELXL97* (Sheldrick, 2008 ▶); molecular graphics: *ORTEP-3* (Farrugia, 1997 ▶) and *DIAMOND* (Brandenburg, 2006 ▶); software used to prepare material for publication: *publCIF* (Westrip, 2010 ▶).

## Supplementary Material

Crystal structure: contains datablock(s) global, I. DOI: 10.1107/S1600536811031953/hb6353sup1.cif

Structure factors: contains datablock(s) I. DOI: 10.1107/S1600536811031953/hb6353Isup2.hkl

Supplementary material file. DOI: 10.1107/S1600536811031953/hb6353Isup3.cml

Additional supplementary materials:  crystallographic information; 3D view; checkCIF report

## Figures and Tables

**Table 1:** Hydrogen-bond geometry (Å, °) *Cg*1 and *Cg*2 are the centroids of the C1—C6 and C17–C22 rings, respectively.

*D*—H⋯*A*	*D*—H	H⋯*A*	*D*⋯*A*	*D*—H⋯*A*
N2—H2⋯N3	0.901 (19)	1.860 (18)	2.5584 (15)	132.8 (16)
N4—H4⋯O1^i^	0.856 (18)	2.089 (19)	2.8946 (14)	156.4 (16)
C2—H2a⋯O3^ii^	0.95	2.38	3.0977 (16)	132
C20—H20⋯*Cg*1^iii^	0.95	2.72	3.4980 (15)	140
C15—H15a⋯*Cg*2^iii^	0.98	2.79	3.4052 (15)	121

Symmetry codes: (i) 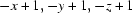; (ii) 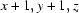; (iii) 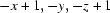.

**Fig. 1.**
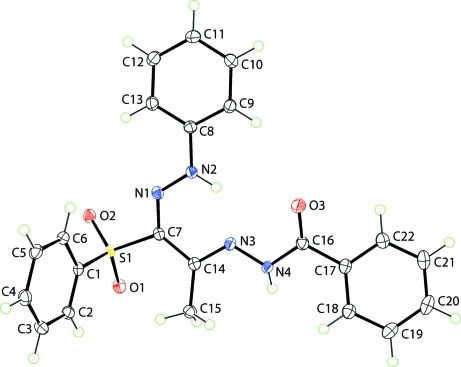
The molecular structure of (I) showing displacement ellipsoids at the 50% probability level.

**Fig. 2.**
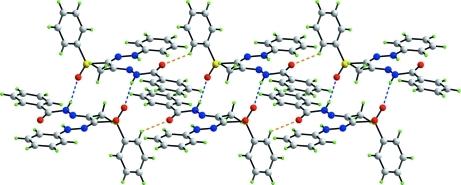
Supramolecular chain in (I) mediated by N—H···O hydrogen bonds (blue dashed lines), leading to centrosymmetric aggregates, and C—H···O interactions, shown as orange lines.

**Fig. 3.**
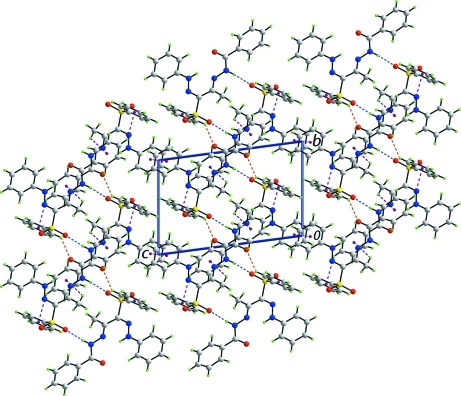
A view in projection down the a axis of the unit-cell contents of (I). The N—H···O, C—H···O, C—H···π and π–π interactions are shown as blue, orange, purple and pink dashed lines, respectively.

## supplementary crystallographic information

### Comment

The title compound (I) was characterized in relation to on-going studies of bis-hydrazone derivatives which have been investigated for biological activity (Abdel-Aziz & Mekawey, 2009; Abdel-Aziz *et al.*, 2010). The molecular structure of (I), Fig. 1, shows an *E* configuration about each of the C═N bonds [C7═N1 = 1.3130 (16) Å and C14═N3 = 1.2936 (17) Å]. The presence of an intramolecular N—H···N hydrogen bond closes a *S*(6) ring, {···HNNCCN}, and provides stabilization to the approximately co-planar arrangement between the phenylhydrazin-1-ylidene and benzohydrazide residues. This co-planarity does not extend to the hydrazine-benzene ring [the N1—N2—C8—C9 torsion angle is -170.74 (11) Å] nor to the benzoyl-benzene [N4—C16—C17—C18 = -21.72 (18) °] ring as significant twists are evident. Nevertheless, to a first approximation the phenylhydrazin-1-ylidene and benzohydrazide residues are co-planar and the sulfonyl-benzene occupies a position almost perpendicular to this plane as seen in the value of the C1—S1—C7—N1 torsion angle of -92.28 (10) °.

In the crystal packing, N—H···O hydrogen bonding between hydrazide-H and a sulfonyl-O leads to the formation of centrosymmetric aggregates *via* a 14-membered {···HNNC_2_SO}_2_ synthon, Table 1. These are connected into a linear supramolecular chain *via* C—H···O interactions, Table 1 and Fig. 2. The chains are connected into layers in the *ac* plane *via* C—H···π interactions, Table 1. Layers are connected along the *b* direction *via*π–π interactions occurring between centrosymmetrically related hydrazine-bound benzene rings [3.7425 (9) Å for symmetry operation: -*x*, -*y*, -*z*].

### Experimental

The title compound was prepared by the literature procedure (Abdel-Aziz *et al.*, 2010) and yellow prisms were isolated from its solution in EtOH/DMF (*v*/*v* = 5/1) by slow evaporation at room temperature.

### Refinement

Carbon-bound H-atoms were placed in calculated positions [C—H 0.95 to 0.98 Å, *U*_iso_(H) 1.2 to 1.5*U*_eq_(C)] and were included in the refinement in the riding model approximation. The amino-H atoms were located in a difference Fourier map, and subsequently refined freely.

### Crystal data

**Table:**

C_22_H_20_N_4_O_3_S	*Z* = 2
*M**_r_* = 420.48	*F*(000) = 440
Triclinic, *P*1	*D*_x_ = 1.398 Mg m^−^^3^
Hall symbol: -P 1	Cu *K*α radiation, λ = 1.5418 Å
*a* = 8.1609 (3) Å	Cell parameters from 4430 reflections
*b* = 9.6632 (5) Å	θ = 3.2–74.2°
*c* = 14.1261 (7) Å	µ = 1.72 mm^−^^1^
α = 92.027 (4)°	*T* = 100 K
β = 102.822 (4)°	Prism, yellow
γ = 111.984 (4)°	0.30 × 0.25 × 0.05 mm
*V* = 998.55 (8) Å^3^	

### Data collection

**Table:**

Agilent SuperNova Dual diffractometer with an Atlas detector	3942 independent reflections
Radiation source: SuperNova (Cu) X-ray Source	3658 reflections with *I* > 2σ(*I*)
mirror	*R*_int_ = 0.017
Detector resolution: 10.4041 pixels mm^-1^	θ_max_ = 74.4°, θ_min_ = 3.2°
ω scans	*h* = −10→8
Absorption correction: multi-scan (*CrysAlis PRO*; Agilent, 2010)	*k* = −12→11
*T*_min_ = 0.825, *T*_max_ = 1.000	*l* = −17→15
6674 measured reflections	

### Refinement

**Table:**

Refinement on *F*^2^	Primary atom site location: structure-invariant direct methods
Least-squares matrix: full	Secondary atom site location: difference Fourier map
*R*[*F*^2^ > 2σ(*F*^2^)] = 0.031	Hydrogen site location: inferred from neighbouring sites
*wR*(*F*^2^) = 0.085	H atoms treated by a mixture of independent and constrained refinement
*S* = 1.04	*w* = 1/[σ^2^(*F*_o_^2^) + (0.0484*P*)^2^ + 0.3295*P*] where *P* = (*F*_o_^2^ + 2*F*_c_^2^)/3
3942 reflections	(Δ/σ)_max_ = 0.001
280 parameters	Δρ_max_ = 0.34 e Å^−^^3^
0 restraints	Δρ_min_ = −0.42 e Å^−^^3^

### Special details

**Table:**

Geometry. All e.s.d.'s (except the e.s.d. in the dihedral angle between two l.s. planes) are estimated using the full covariance matrix. The cell e.s.d.'s are taken into account individually in the estimation of e.s.d.'s in distances, angles and torsion angles; correlations between e.s.d.'s in cell parameters are only used when they are defined by crystal symmetry. An approximate (isotropic) treatment of cell e.s.d.'s is used for estimating e.s.d.'s involving l.s. planes.
Refinement. Refinement of *F*^2^ against ALL reflections. The weighted *R*-factor *wR* and goodness of fit *S* are based on *F*^2^, conventional *R*-factors *R* are based on *F*, with *F* set to zero for negative *F*^2^. The threshold expression of *F*^2^ > σ(*F*^2^) is used only for calculating *R*-factors(gt) *etc*. and is not relevant to the choice of reflections for refinement. *R*-factors based on *F*^2^ are statistically about twice as large as those based on *F*, and *R*- factors based on ALL data will be even larger.

### Fractional atomic coordinates and isotropic or equivalent isotropic displacement parameters (Å^2^)

**Table:**

	*x*	*y*	*z*	*U*_iso_*/*U*_eq_	
S1	0.50233 (4)	0.56979 (3)	0.25827 (2)	0.01376 (10)	
O1	0.58750 (13)	0.66428 (10)	0.35165 (7)	0.0188 (2)	
O2	0.38284 (12)	0.61117 (10)	0.18465 (7)	0.0184 (2)	
O3	0.10801 (14)	−0.07033 (11)	0.38899 (7)	0.0272 (2)	
N1	0.24156 (14)	0.31176 (12)	0.20702 (8)	0.0156 (2)	
N2	0.12879 (15)	0.17365 (12)	0.20937 (8)	0.0162 (2)	
H2	0.146 (2)	0.134 (2)	0.2655 (14)	0.031 (5)*	
N3	0.31255 (14)	0.20873 (12)	0.38694 (8)	0.0157 (2)	
N4	0.34253 (15)	0.14291 (12)	0.46998 (8)	0.0155 (2)	
H4	0.393 (2)	0.196 (2)	0.5262 (13)	0.024 (4)*	
C1	0.67600 (17)	0.55617 (14)	0.20755 (9)	0.0142 (2)	
C2	0.85772 (17)	0.62654 (14)	0.25910 (9)	0.0172 (3)	
H2A	0.8906	0.6826	0.3218	0.021*	
C3	0.99099 (18)	0.61328 (15)	0.21697 (10)	0.0202 (3)	
H3	1.1162	0.6608	0.2512	0.024*	
C4	0.94206 (18)	0.53115 (16)	0.12537 (10)	0.0204 (3)	
H4A	1.0337	0.5217	0.0975	0.025*	
C5	0.75863 (18)	0.46236 (15)	0.07400 (9)	0.0190 (3)	
H5	0.7258	0.4071	0.0110	0.023*	
C6	0.62441 (17)	0.47453 (14)	0.11483 (9)	0.0162 (3)	
H6	0.4993	0.4281	0.0803	0.019*	
C7	0.38026 (17)	0.38545 (14)	0.28212 (9)	0.0147 (2)	
C8	−0.02363 (17)	0.09899 (14)	0.12996 (9)	0.0157 (3)	
C9	−0.15086 (18)	−0.03770 (14)	0.14394 (9)	0.0183 (3)	
H9	−0.1313	−0.0773	0.2041	0.022*	
C10	−0.30598 (19)	−0.11519 (15)	0.06928 (10)	0.0210 (3)	
H10	−0.3932	−0.2084	0.0783	0.025*	
C11	−0.33482 (19)	−0.05745 (16)	−0.01863 (10)	0.0224 (3)	
H11	−0.4418	−0.1105	−0.0695	0.027*	
C12	−0.2058 (2)	0.07881 (16)	−0.03178 (10)	0.0228 (3)	
H12	−0.2252	0.1180	−0.0921	0.027*	
C13	−0.04996 (18)	0.15771 (15)	0.04185 (10)	0.0188 (3)	
H13	0.0377	0.2504	0.0325	0.023*	
C14	0.43798 (17)	0.33214 (14)	0.37516 (9)	0.0147 (2)	
C15	0.62412 (17)	0.40633 (14)	0.44494 (9)	0.0173 (3)	
H15A	0.6634	0.3290	0.4728	0.026*	
H15B	0.6187	0.4718	0.4978	0.026*	
H15C	0.7114	0.4665	0.4098	0.026*	
C16	0.21874 (18)	−0.00233 (14)	0.46522 (9)	0.0171 (3)	
C17	0.23417 (18)	−0.07141 (14)	0.55775 (9)	0.0166 (3)	
C18	0.39363 (19)	−0.02070 (15)	0.63346 (10)	0.0190 (3)	
H18	0.4977	0.0635	0.6283	0.023*	
C19	0.4000 (2)	−0.09350 (16)	0.71628 (10)	0.0236 (3)	
H19	0.5090	−0.0598	0.7676	0.028*	
C20	0.2473 (2)	−0.21564 (16)	0.72444 (10)	0.0247 (3)	
H20	0.2516	−0.2643	0.7816	0.030*	
C21	0.0886 (2)	−0.26655 (16)	0.64928 (11)	0.0231 (3)	
H21	−0.0154	−0.3503	0.6549	0.028*	
C22	0.08182 (18)	−0.19513 (15)	0.56582 (10)	0.0195 (3)	
H22	−0.0267	−0.2305	0.5141	0.023*	

### Atomic displacement parameters (Å^2^)

**Table:**

	*U*^11^	*U*^22^	*U*^33^	*U*^12^	*U*^13^	*U*^23^
S1	0.01522 (16)	0.01194 (15)	0.01440 (16)	0.00461 (12)	0.00554 (11)	0.00199 (11)
O1	0.0231 (5)	0.0151 (4)	0.0175 (4)	0.0056 (4)	0.0075 (4)	−0.0008 (3)
O2	0.0183 (4)	0.0180 (4)	0.0211 (5)	0.0086 (4)	0.0063 (4)	0.0066 (4)
O3	0.0318 (6)	0.0185 (5)	0.0178 (5)	−0.0002 (4)	−0.0017 (4)	0.0018 (4)
N1	0.0164 (5)	0.0142 (5)	0.0161 (5)	0.0047 (4)	0.0061 (4)	0.0012 (4)
N2	0.0176 (5)	0.0140 (5)	0.0140 (5)	0.0028 (4)	0.0039 (4)	0.0020 (4)
N3	0.0182 (5)	0.0158 (5)	0.0136 (5)	0.0067 (4)	0.0049 (4)	0.0036 (4)
N4	0.0185 (5)	0.0142 (5)	0.0114 (5)	0.0044 (4)	0.0027 (4)	0.0020 (4)
C1	0.0167 (6)	0.0124 (6)	0.0147 (6)	0.0053 (5)	0.0063 (5)	0.0048 (5)
C2	0.0182 (6)	0.0151 (6)	0.0153 (6)	0.0035 (5)	0.0039 (5)	0.0014 (5)
C3	0.0145 (6)	0.0206 (6)	0.0229 (7)	0.0037 (5)	0.0046 (5)	0.0046 (5)
C4	0.0203 (6)	0.0234 (7)	0.0215 (7)	0.0097 (5)	0.0103 (5)	0.0069 (5)
C5	0.0228 (7)	0.0206 (7)	0.0141 (6)	0.0082 (5)	0.0060 (5)	0.0026 (5)
C6	0.0162 (6)	0.0163 (6)	0.0150 (6)	0.0054 (5)	0.0031 (5)	0.0036 (5)
C7	0.0154 (6)	0.0131 (6)	0.0155 (6)	0.0044 (5)	0.0060 (5)	0.0018 (5)
C8	0.0168 (6)	0.0146 (6)	0.0147 (6)	0.0057 (5)	0.0036 (5)	−0.0002 (5)
C9	0.0212 (6)	0.0170 (6)	0.0153 (6)	0.0059 (5)	0.0042 (5)	0.0035 (5)
C10	0.0198 (6)	0.0173 (6)	0.0208 (7)	0.0021 (5)	0.0045 (5)	0.0016 (5)
C11	0.0214 (7)	0.0211 (7)	0.0179 (6)	0.0042 (5)	−0.0005 (5)	0.0003 (5)
C12	0.0270 (7)	0.0222 (7)	0.0169 (6)	0.0084 (6)	0.0029 (5)	0.0047 (5)
C13	0.0215 (6)	0.0157 (6)	0.0176 (6)	0.0050 (5)	0.0060 (5)	0.0031 (5)
C14	0.0162 (6)	0.0144 (6)	0.0147 (6)	0.0065 (5)	0.0054 (5)	0.0007 (5)
C15	0.0170 (6)	0.0169 (6)	0.0175 (6)	0.0060 (5)	0.0046 (5)	0.0014 (5)
C16	0.0187 (6)	0.0147 (6)	0.0168 (6)	0.0054 (5)	0.0046 (5)	0.0016 (5)
C17	0.0212 (6)	0.0147 (6)	0.0169 (6)	0.0091 (5)	0.0068 (5)	0.0029 (5)
C18	0.0227 (7)	0.0171 (6)	0.0180 (6)	0.0085 (5)	0.0058 (5)	0.0024 (5)
C19	0.0311 (7)	0.0259 (7)	0.0167 (6)	0.0155 (6)	0.0038 (5)	0.0034 (5)
C20	0.0389 (8)	0.0258 (7)	0.0202 (7)	0.0203 (6)	0.0143 (6)	0.0102 (6)
C21	0.0283 (7)	0.0202 (7)	0.0297 (7)	0.0128 (6)	0.0175 (6)	0.0102 (6)
C22	0.0206 (6)	0.0179 (6)	0.0230 (7)	0.0090 (5)	0.0087 (5)	0.0046 (5)

### Geometric parameters (Å, °)

**Table:**

S1—O2	1.4365 (9)	C8—C9	1.3945 (18)
S1—O1	1.4456 (9)	C9—C10	1.3847 (18)
S1—C1	1.7672 (13)	C9—H9	0.9500
S1—C7	1.7725 (13)	C10—C11	1.3880 (19)
O3—C16	1.2172 (16)	C10—H10	0.9500
N1—C7	1.3130 (16)	C11—C12	1.394 (2)
N1—N2	1.3139 (15)	C11—H11	0.9500
N2—C8	1.4062 (16)	C12—C13	1.3826 (19)
N2—H2	0.901 (19)	C12—H12	0.9500
N3—C14	1.2936 (17)	C13—H13	0.9500
N3—N4	1.3720 (15)	C14—C15	1.5044 (17)
N4—C16	1.3771 (16)	C15—H15A	0.9800
N4—H4	0.856 (18)	C15—H15B	0.9800
C1—C2	1.3866 (18)	C15—H15C	0.9800
C1—C6	1.3960 (18)	C16—C17	1.4905 (17)
C2—C3	1.3927 (19)	C17—C18	1.3947 (18)
C2—H2A	0.9500	C17—C22	1.3956 (18)
C3—C4	1.3872 (19)	C18—C19	1.3875 (19)
C3—H3	0.9500	C18—H18	0.9500
C4—C5	1.3957 (19)	C19—C20	1.390 (2)
C4—H4A	0.9500	C19—H19	0.9500
C5—C6	1.3849 (18)	C20—C21	1.386 (2)
C5—H5	0.9500	C20—H20	0.9500
C6—H6	0.9500	C21—C22	1.3881 (19)
C7—C14	1.4729 (17)	C21—H21	0.9500
C8—C13	1.3928 (18)	C22—H22	0.9500
			
O2—S1—O1	118.61 (6)	C9—C10—H10	119.8
O2—S1—C1	107.72 (6)	C11—C10—H10	119.8
O1—S1—C1	108.38 (6)	C10—C11—C12	119.60 (12)
O2—S1—C7	108.78 (6)	C10—C11—H11	120.2
O1—S1—C7	107.22 (6)	C12—C11—H11	120.2
C1—S1—C7	105.38 (6)	C13—C12—C11	120.86 (12)
C7—N1—N2	121.00 (11)	C13—C12—H12	119.6
N1—N2—C8	120.16 (11)	C11—C12—H12	119.6
N1—N2—H2	117.7 (11)	C12—C13—C8	118.89 (12)
C8—N2—H2	121.8 (11)	C12—C13—H13	120.6
C14—N3—N4	119.70 (11)	C8—C13—H13	120.6
N3—N4—C16	114.32 (10)	N3—C14—C7	111.66 (11)
N3—N4—H4	120.7 (12)	N3—C14—C15	123.98 (11)
C16—N4—H4	118.2 (12)	C7—C14—C15	124.24 (11)
C2—C1—C6	121.75 (12)	C14—C15—H15A	109.5
C2—C1—S1	120.14 (10)	C14—C15—H15B	109.5
C6—C1—S1	118.11 (9)	H15A—C15—H15B	109.5
C1—C2—C3	118.60 (12)	C14—C15—H15C	109.5
C1—C2—H2A	120.7	H15A—C15—H15C	109.5
C3—C2—H2A	120.7	H15B—C15—H15C	109.5
C4—C3—C2	120.42 (12)	O3—C16—N4	121.44 (12)
C4—C3—H3	119.8	O3—C16—C17	122.71 (12)
C2—C3—H3	119.8	N4—C16—C17	115.81 (11)
C3—C4—C5	120.25 (12)	C18—C17—C22	119.76 (12)
C3—C4—H4A	119.9	C18—C17—C16	122.99 (12)
C5—C4—H4A	119.9	C22—C17—C16	117.22 (12)
C6—C5—C4	120.05 (12)	C19—C18—C17	119.85 (13)
C6—C5—H5	120.0	C19—C18—H18	120.1
C4—C5—H5	120.0	C17—C18—H18	120.1
C5—C6—C1	118.92 (12)	C18—C19—C20	120.18 (13)
C5—C6—H6	120.5	C18—C19—H19	119.9
C1—C6—H6	120.5	C20—C19—H19	119.9
N1—C7—C14	128.30 (11)	C21—C20—C19	120.14 (13)
N1—C7—S1	110.37 (9)	C21—C20—H20	119.9
C14—C7—S1	121.33 (9)	C19—C20—H20	119.9
C13—C8—C9	120.90 (12)	C20—C21—C22	120.00 (13)
C13—C8—N2	122.56 (11)	C20—C21—H21	120.0
C9—C8—N2	116.54 (11)	C22—C21—H21	120.0
C10—C9—C8	119.36 (12)	C21—C22—C17	120.06 (13)
C10—C9—H9	120.3	C21—C22—H22	120.0
C8—C9—H9	120.3	C17—C22—H22	120.0
C9—C10—C11	120.39 (12)		
			
C7—N1—N2—C8	177.15 (11)	N2—C8—C9—C10	178.61 (12)
C14—N3—N4—C16	−166.62 (11)	C8—C9—C10—C11	−0.1 (2)
O2—S1—C1—C2	131.46 (10)	C9—C10—C11—C12	0.5 (2)
O1—S1—C1—C2	1.97 (12)	C10—C11—C12—C13	−0.4 (2)
C7—S1—C1—C2	−112.54 (11)	C11—C12—C13—C8	−0.1 (2)
O2—S1—C1—C6	−47.94 (11)	C9—C8—C13—C12	0.6 (2)
O1—S1—C1—C6	−177.43 (9)	N2—C8—C13—C12	−178.46 (12)
C7—S1—C1—C6	68.06 (11)	N4—N3—C14—C7	−179.77 (10)
C6—C1—C2—C3	−0.71 (19)	N4—N3—C14—C15	3.87 (18)
S1—C1—C2—C3	179.92 (10)	N1—C7—C14—N3	−13.99 (19)
C1—C2—C3—C4	0.0 (2)	S1—C7—C14—N3	166.60 (9)
C2—C3—C4—C5	0.7 (2)	N1—C7—C14—C15	162.36 (12)
C3—C4—C5—C6	−0.7 (2)	S1—C7—C14—C15	−17.05 (17)
C4—C5—C6—C1	−0.05 (19)	N3—N4—C16—O3	8.74 (18)
C2—C1—C6—C5	0.75 (19)	N3—N4—C16—C17	−173.41 (10)
S1—C1—C6—C5	−179.86 (10)	O3—C16—C17—C18	156.10 (13)
N2—N1—C7—C14	2.3 (2)	N4—C16—C17—C18	−21.72 (18)
N2—N1—C7—S1	−178.25 (9)	O3—C16—C17—C22	−21.95 (19)
O2—S1—C7—N1	22.99 (11)	N4—C16—C17—C22	160.23 (12)
O1—S1—C7—N1	152.40 (9)	C22—C17—C18—C19	0.04 (19)
C1—S1—C7—N1	−92.28 (10)	C16—C17—C18—C19	−177.96 (12)
O2—S1—C7—C14	−157.51 (10)	C17—C18—C19—C20	−0.7 (2)
O1—S1—C7—C14	−28.10 (11)	C18—C19—C20—C21	0.9 (2)
C1—S1—C7—C14	87.22 (11)	C19—C20—C21—C22	−0.3 (2)
N1—N2—C8—C13	8.35 (19)	C20—C21—C22—C17	−0.4 (2)
N1—N2—C8—C9	−170.74 (11)	C18—C17—C22—C21	0.54 (19)
C13—C8—C9—C10	−0.5 (2)	C16—C17—C22—C21	178.65 (12)

### Hydrogen-bond geometry (Å, °)

**Table:**

Cg1 and Cg2 are the centroids of the C1—C6 and C17–C22 rings, respectively.

**Table:**

*D*—H···*A*	*D*—H	H···*A*	*D*···*A*	*D*—H···*A*
N2—H2···N3	0.901 (19)	1.860 (18)	2.5584 (15)	132.8 (16)
N4—H4···O1^i^	0.856 (18)	2.089 (19)	2.8946 (14)	156.4 (16)
C2—H2a···O3^ii^	0.95	2.38	3.0977 (16)	132
C20—H20···Cg1^iii^	0.95	2.72	3.4980 (15)	140
C15—H15a···Cg2^iii^	0.98	2.79	3.4052 (15)	121

Symmetry codes: (i) −*x*+1, −*y*+1, −*z*+1; (ii) *x*+1, *y*+1, *z*; (iii) −*x*+1, −*y*, −*z*+1.

## References

[bb1] Abdel-Aziz, H. A., Abdel-Wahab, B. F. & Badria, F. A. (2010). *Arch. Pharm.* **343**, 152–159. 10.1002/ardp.200900195 20186867

[bb2] Abdel-Aziz, H. A. & Mekawey, A. A. I. (2009). *Eur. J. Med. Chem.* **44**, 3985–4997. 10.1016/j.ejmech.2009.09.002 19782439

[bb3] Agilent (2010). *CrysAlis PRO* Agilent Technologies, Yarnton, England.

[bb4] Brandenburg, K. (2006). *DIAMOND* Crystal Impact GbR, Bonn, Germany.

[bb5] Farrugia, L. J. (1997). *J. Appl. Cryst.* **30**, 565.

[bb6] Sheldrick, G. M. (2008). *Acta Cryst.* A**64**, 112–122. 10.1107/S0108767307043930 18156677

[bb7] Westrip, S. P. (2010). *J. Appl. Cryst.* **43**, 920–925.

